# Supplementary material for: Psychological Features of Fibromyalgia in the Psychological Health Services
Source: Behav Sci (Basel). 2024 Nov 1;14(11):1016. doi: 10.3390/bs14111016 (PMC11591246; doi:10.3390/bs14111016)
Supplement: Supplementary file 1 [file behavsci-14-01016-s001.zip › behavsci-3091571-supplementary.pdf]

Supplementary Materials

Psychological Features of Fibromyalgia in the Psychological Health Services

Gabriela Rios Andreghetti [1,†], Sonia Montemurro [1,†], Luca Rizzi [2], Laura Casetta [2], Marcello Passarelli [3], Sara Mondini [1,4,5,6,7,*,‡] and Diego Rocco [4,‡]

[1] Department of Philosophy, Sociology, Education and Applied Psychology (FISPPA), University of Padua, 35122 Padua, Italy; gabriela.riosandreghetti@studenti.unipd.it (G.R.A.); sonia.montemurro@unipd.it (S.M.)
[2] Associazione Centro di Psicologia e Psicoterapia Funzionale, Istituto SIF di Padova, 35138 Padua, Italy; luca.rizzi@ymail.com (L.R.); laura.casetta@yahoo.it (L.C.)
[3] Institute of Educational Technology, National Research Council, 16149 Genoa, Italy; marcello.passarelli@cnr.it
[4] Department of Developmental Psychology and Socialization (DPSS), University of Padua, 35131 Padua, Italy; diego.rocco@unipd.it
[5] Human Inspired Technology—Research Centre HIT, University of Padua, 35122 Padua, Italy
[6] Servizi Clinici Universitari Psicologici (SCUP)—Centro di Ateneo, University of Padua, 35131 Padua, Italy
[7] IRCCS San Camillo Hospital, 30126 Venice, Italy
* Correspondence: sara.mondini@unipd.it
† These authors contributed equally to this work and share the first Co-authorship.
‡ These authors contributed equally to this work and share the last Co-authorship.

Characteristics of the Sample		
	N° of participants	Description
Geographical origins	11	Lived in the same region where the research was carried out
	57	Lived in different Italian regions
	8	Did not answer
Relational Status	46	Married
	12	Involved in a stable relationship
	9	Divorced
	7	Single
	1	Widow
	1	Did not answer
Education	4	Specialization, e.g., PhD/MD
	17	Master's Degree
	6	Bachelor’s Degree
	28	High School Degree
	21	Middle School Degree
Occupational Status	42	Employed
	12	Unemployed
	5	Housewives
	5	Retired

	2	Students
	11	Did not reply
Psychotherapy	19	Undergoing Psychotherapy
	38	Had undergone Psychotherapy (at least once?) in the past
Diagnosis of Fibromyalgia	13	≤ 1 year
	24	2 - 5 years
	21	6 - 10 years
	18	> 18 years
Medical Treatment for Fibromyalgia	54	Ongoing pharmacological treatment
	22	No pharmacological treatment

Table S1. Characteristics of the sample. The first column refers to the variable considered, the second column to the number of people who responded, the third to the measurement.

Scale/ Subscale	Mean ± SD	Median	Min	Max	Normative data population	Normative data mean ± SD
**TAQ**						
TAQ – Partner	3.55 ± .71	3.50	1.80	5.0		1.97 ± 0.63
TAQ – Family	3.01 ± .97	3.00	1.17	5.0		2.60 ± 0.99
TAQ – Same Sex	4.02 ±.70	4.08	2.17	5.0	Italian healthy women (N = 217)	1.86 ± 0.60
TAQ - Opposite Sex	3.62 ±.85	3.50	1.00	5.0		2.18 ± 0.79
TAQ - Stranger	3.17 ± .87	3.00	1.00	5.0		2.61 ± .80
**STAI Y-2**	56.49 ± 10.80	58.50	32.00	78.00	Italian healthy women (N = 105)	41.8 ± 10
**BDI-II**	19.38 ± 7.41	18.00	0.28	1.76	Italian healthy women (N = 380)	9.5 ± 5.5
**iPQ-16**	7.32 ± 3.56	7.00	0	14	Italian help-seeking young adults not at high risk (N = 81)	4.19 ± 3.48
**FSCRS**						
FSCRS – Hated-Self	1.83 ± .72	1.60	1.00	4.40		1.6 ±.7
FSCRS – Inadequate-Self	2.85 ± .76	2.78	1.11	4.78	Italian population (N = 539)	2.7 ± .9
FSCRS – Reassured-Self	3.03 ± .70	2.94	1.37	4.75		3.5 ±.7
**CPAQ**						
CPAQ - Pain Willingness	19.11 ± 8.75	18.00	2.00	45.00		22.1 ± 10.0

Scale/ Subscale	Mean ± SD	Median	Min	Max	Normative data population	Normative data mean ± SD
CPAQ – Activity Engagement	30.22 ± 11.40	31.00	6.00	51.00	Italian patients with chronic pain (N = 120)	29.4 ± 12.9
CPAQ – Total Score	49.33 ± 17.38	46.50	12.00	95.00		51.5 ± 18.5
**FIQ**						
FIQ – Poor Functioning	4.17 ± 2.14	4.33	0.00	8.67	Italian patients with fibromyalgia (N = 50)	4.2 ± 2.1
FIQ – Total Score	66.55 ± 12.86	66.67	21.86	90.90		57.2 ± 12.8
**DPES**						
DPES – Happiness	3.64 ± 1.23	3.50	1.54	6.00	Italian healthy women (N = 439)	4.63 ±1.00
DPES – Pride	4.54 ±1.24	4.70	1.60	7.00		5.10 ±.86
DPES – Love	4.14 ± 1.17	4.17	1.50	6.17		4.39 ±1.03
DPES – Compassion	6.23 ± .69	6.20	4.40	7.00		5.76 ±.79
DPES – Amusement	4.42 ± 1.40	4.40	1.00	6.80		4.93 ±1.00
DPES – Awe	4.51 ± 1.30	4.60	1.00	6.80		4.55 ± 1.03

Table S2. Descriptive analyses of the fibromyalgia and normative groups. The means, medians, standard deviations and ranges of each variable considered in the subsequent analyses are shown in the table. Normative means and standard deviations are reported together with information about the normative population.

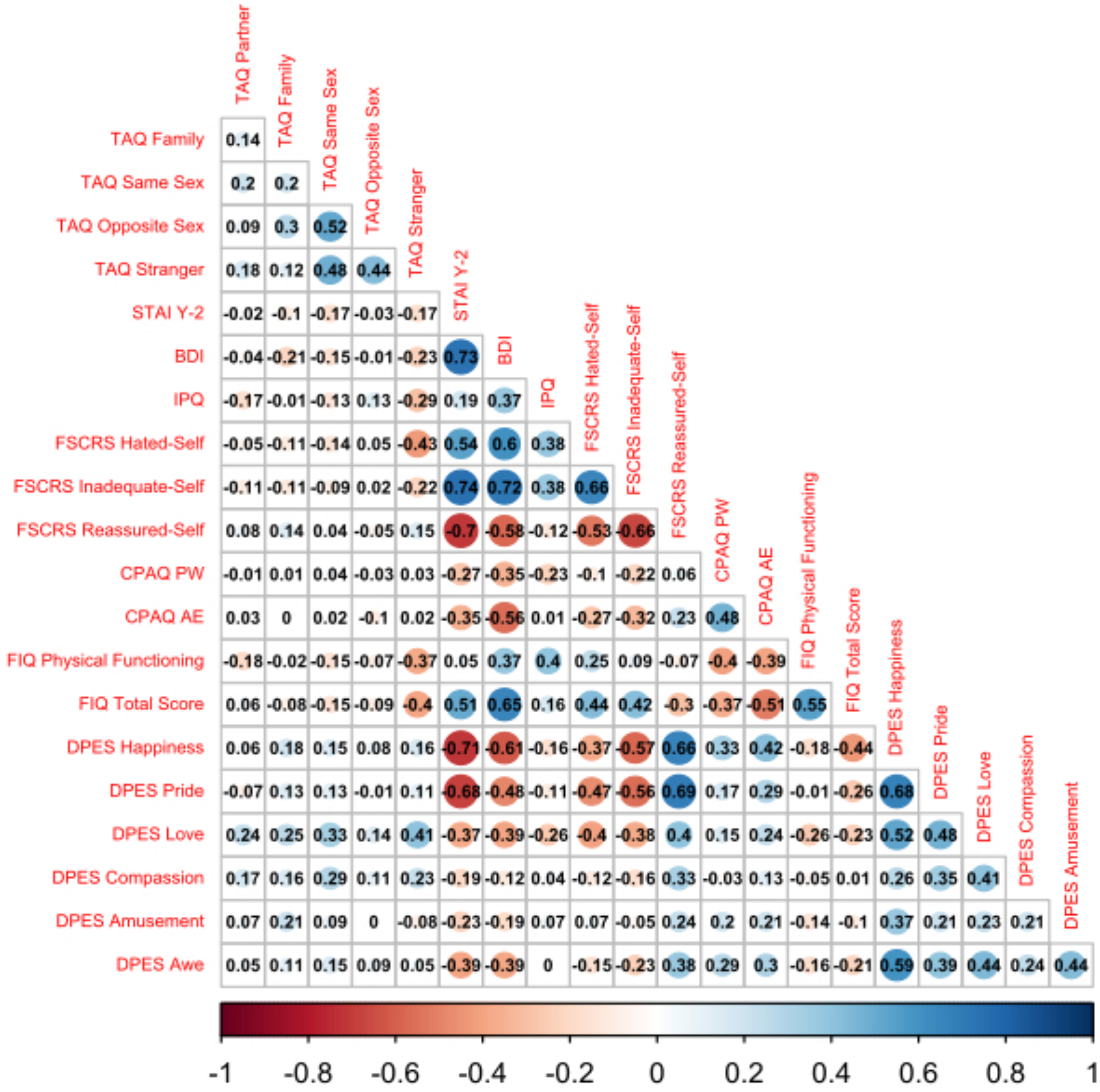

Figure S1. Correlation plot of subscale scores.
